# Are female daycare workers at greater risk of cytomegalovirus infection? A secondary data analysis of CMV seroprevalence between 2010 and 2013 in Hamburg, Germany

**DOI:** 10.3205/dgkh000269

**Published:** 2016-04-21

**Authors:** Johanna Stranzinger, Agnessa Kozak, Benjamin Schilgen, Diana Paris, Thomas Nießen, Lutz Schmidt, Andreas Wille, Norbert L. Wagner, Albert Nienhaus

**Affiliations:** 1 Institute for Statutory Accident Insurance and Prevention in the Health and Welfare Services, Hamburg, Germany; 2 University Medical Center Hamburg-Eppendorf, Institute for Health Services Research in Dermatology and Nursing, Hamburg, Germany; 3 Department for Occupational Health Service of the Administration of Hamburg, Hamburg, Germany; 4 Central Institute for Transfusion Medicine, Hamburg, Germany; 5 State Institute for Food Safety, Health and Environment, Hamburg, Germany; 6 Saw Swee Hock School of Public Health, National University of Singapore, Singapore

**Keywords:** daycare workers (DCWs), daycare providers, nursery educators, CMV infection, female blood donors

## Abstract

**Background:** Close contact with asymptomatic children younger than three years is a risk factor for a primary cytomegalovirus (CMV) infection. In pregnant women, such primary infection increases the risk of CMV-induced feto- or embryopathy. Daycare providers have therefore implemented working restrictions for pregnant daycare workers (DCWs) in accordance with legislation and guidelines for maternity protection. However, little is known about the infection risk for DCWs. We therefore compared the prevalence of CMV antibodies of pregnant DCWs to that of female blood donors (BDs).

**Method:** In a secondary data analysis, the prevalence of anti-CMV IgG among pregnant DCWs (N=509) in daycare centers (DCCs) was compared to the prevalence of female first-time BDs (N=14,358) from the greater region of Hamburg, Germany. Data collection took place between 2010 and 2013. The influence of other risk factors such as age, pregnancies and place of residence was evaluated using logistic regression models.

**Results:** The prevalence of CMV antibodies in pregnant DCWs was higher than in female BDs (54.6 vs 41.5%; OR 1.6; 95%CI 1.3–1.9). The subgroup of BDs who had given birth to at least one child and who lived in the city of Hamburg (N=2,591) had a prevalence of CMV antibodies similar to the prevalence in pregnant DCWs (53.9 vs 54.6%; OR 0.9; 95%CI 0.8–1.2). Age, pregnancy history and living in the center of Hamburg were risk factors for CMV infections.

**Conclusion:** The comparison of pregnant DCWs to the best-matching subgroup of female first-time BDs with past pregnancies and living in the city of Hamburg does not indicate an elevated risk of CMV infection among DCWs. However, as two secondary data sets from convenience samples were used, a more detailed investigation of the risk factors other than place of residence, age and maternity was not possible. Therefore, the CMV infection risk in DCWs should be further studied by taking into consideration the potential preventive effect of hygiene measures.

## Zusammenfassung

**Hintergrund:** Enger Kontakt zu asymptomatischen Kindern unter drei Jahren gilt als Risikofaktor für eine primäre Cytomegalievirus (CMV)-Infektion. Eine Primärinfektion während der Schwangerschaft kann zu einer CMV bedingten Feto- und Embryopathie führen. In Übereinstimmung mit dem Mutterschutzgesetz gibt es daher Tätigkeitsbeschränkungen für schwangere Erzieherinnen in Kindertagesstätten (KiTa), die Anti-CMV negativ sind. Bisher ist jedoch wenig über das tatsächliche Infektionsrisiko in KiTas bekannt. Wir haben deshalb die Prävalenz von CMV-Antikörpern bei schwangeren Erzieherinnen mit derjenigen von Blutspenderinnen verglichen.

**Methoden:** In einer Gelegenheitsdatenanalyse wurde die Prävalenz von Anti-CMV IgG bei schwangeren Erzieherinnen von KiTas (N=509) mit derjenigen von neuen Blutspenderinnen (n=14,358) aus Hamburg und Umgebung verglichen. Die Daten wurden zwischen 2010 und 2013 erhoben. Der Einfluss anderer Risikofaktoren wie Alter, Schwangerschaft und Wohnort wurde mittels logistischer Regression überprüft.

**Ergebnisse:** Schwangere Erzieherinnen hatten eine höhere CMV-Antikörper-Prävalenz als Blutspenderinnen (54,6 vs. 41,5%; OR 1,6; 95%CI 1,3–1.9). Blutspenderinnen mit mindestens einem Kind und Wohnort in Hamburg (n=2,591) hatten eine ähnlich hohe Prävalenz wie die Erzieherinnen (53,9 vs. 54,6%; OR 0,9; 95%CI 0,8–1.2). Alter, Schwangerschaften und Wohnort in Hamburg waren Risikofaktoren für eine CMV-Infektion.

**Schlussfolgerungen:** Der Vergleich mit der wahrscheinlich am besten geeigneten Gruppe ergab kein erhöhtes Risiko für CMV-Infektionen bei Erzieherinnen in KiTas. Da jedoch lediglich Gelegenheitsdaten für die sekundäre Datenanalyse verwendet wurden, sollte das Infektionsrisiko für Erzieherinnen unter Berücksichtigung von möglichen Risikofaktoren genauer untersucht werden. Ferner sollte der protektive Effekt von Präventionsmaßnahmen untersucht werden.

## Background

A primary cytomegalovirus (CMV) infection during pregnancy increases the risk of congenital anomalies, while this risk seems to be minor for secondary infections during pregnancy [1], [2], [3], [4], [5], [6], [7], [8]. Infections occur in all age groups [9]. CMV enters latency following primary infection and can subsequently reactivate. Reinfection with a different viral strain can also occur. As CMV is shed in bodily fluids, risk factors for transmission are intimate contact, being breastfed, care of small children, as well as low educational level, hygienic and socioeconomic standards [9], [10], [11], [12], [13], [14]. Why is contact with little children a key risk factor? Half of breastfeeding mothers are healthy CMV carriers and share the viruses with their babies via lactation. Approximately one-third of breastfed children become asymptomatic CMV carriers themselves for months or years with the potential to infect or reinfect others. 

Based on the German Maternity Protection Law (Mutterschutzgesetz), German guidelines on maternity protection therefore demand restrictions concerning work for pregnant anti-CMV-negative daycare workers (DCWs). In order to protect DCWs from primary infection, their CMV serostatus must be checked at the beginning of their pregnancy. When the DCW is seronegative, she is excluded from professional activities with children under the age of three years in order to prevent feto- or embryopathy in her offspring. Given the shortage of DCWs, these restrictions might pose problems for some daycare centers (DCCs). As studies on CMV infection rates in DCWs are lacking, we analyzed data relating to pregnant DCWs and blood donors (BDs) in the same geographical region to provide an initial overview of the prevalence of CMV infections in DCWs in comparison with the general population. 

## Methods

We examined two anonymized data sets. The first data set comprised pregnant DCWs (N=517) living in Hamburg, and the second data set comprised female first-time BDs (N=16,286). Both samples were collected between 2010 and 2013 in the geographical region of the city of Hamburg, Germany, and its surrounding districts. Information included date of birth, date of blood sample, and gender. Information about pregnancies and place of work or residence differed in both samples. In contrast to the DCWs, BDs were not knowingly pregnant at the time of sampling, but had a medical record with information about past pregnancies. Furthermore, place of residence was determined by postal codes. In both groups – DCWs and BDs – specific immunoglobulin antibodies (anti-CMV IgG) were analyzed using the enzyme-linked immunosorbent assay (ELISA). Seropositivity was defined as the stable presence of anti-CMV IgG. 

Data relating to DCWs came from the State Institute for Food Safety, Health and Environment, Hamburg. The blood samples were taken during a medical examination by a company doctor at the beginning of the pregnancy. This dataset contained information on place of work, age, and CMV serostatus. The data was double-checked by the company doctor to verify the identity of participants with identical dates of birth. If a DCW was examined twice, only the results related to the first pregnancy were considered. The analysis was limited to female DCWs younger than 45 years of age, as only eight DCWs were older than 45 years. The test results of 509 DCWs were therefore analyzed.

The dataset comprising BDs was provided by the Central Institute for Transfusion Medicine, Hamburg. Originally, it included the results of CMV IgG tests of 16,286 first-time BDs, age, information about past pregnancies (“Have you ever been pregnant?”) and place of current residence specified by postal code. Occupation was documented in a non-standardized way; therefore we refrained from including it in our analysis. We excluded 16 cases due to a lack of data on CM serostatus, and eleven cases due to a lack of information on pregnancies. Furthermore, in order to enable better comparability, 1,901 cases were excluded because they were older than 45 years. The test results of 14,358 female BDs were thus analyzed. 

We examined the relevance of region of residence as a proxy of socioeconomic status (SES). Residence in the city (post codes 20–22) is assumed to be associated with lower SES. We analyzed the data using the following groups:

- BD – Blood donors
- BDPcity – Blood donors with pregnancy, living in the city

## Statistical analyses

Differences between the two groups were examined using contingency table analyses with Pearson’s chi-square test. For ordinal data, the proportions of anti-CMV IgG test results were compared using the chi-square test for trend. Statistical significance was set at p<0.05. Adjusted odds ratios (OR) for anti-CMV IgG test results depending on the available putative risk factors were calculated using logistic regression. A backwards stepwise method was applied for model building using the change criterion [15].

## Ethical consideration

In accordance with the Professional Code for Physicians in Hamburg (Art. 15, 1., as of 10.03.2014) and the Chamber Legislation for Medical Professions in the Federal State of Hamburg (HmbKGH), it is only necessary to obtain advice on questions of professional ethics and professional conduct from an Ethics Committee if data which can be traced to a particular individual are used in a research project. Laboratory data were collected routinely. Both datasets were made available to the research center in an anonymized format. Therefore, no ethical approval was obtained. 

## Results

A total of 509 pregnant DCWs and 14,358 female first-time BDs were eligible for the analysis. The characteristics of both samples are listed in Table 1 (Tab. 1). The DCWs were older than the BDs (mean age 30.7 [SD 4.7] vs 26.6 [SD 7.2]; p<0.001). In particular, BDs were more often younger than 25 years (54.5 vs 12.4%). The majority of BDs resided in the city of Hamburg (94%). They significantly more often tested positive for anti-CMV IgG than those who lived in the surrounding region (42.0 vs 34.2%; p<0.001; Table 2 (Tab. 2)). Around 19.4% of BDs had been pregnant at least once (Table 1 (Tab. 1)). BDs with a history of pregnancy significantly more often tested positive for anti-CMV IgG than those without (53.1 vs 38.7%; p<0.001; Table 2 (Tab. 2)). Compared to the youngest age group, the prevalence of CMV antibodies was slightly increased in all other age groups; however, no clear increase across the different age groups was apparent in the combined dataset (OR between 1.2 and 1.3; Table 3 (Tab. 3)).

Compared to BDs as a whole, the prevalence of anti-CMV IgG among female DCWs was significantly higher (41.5 vs 54.6%. The age-adjusted OR was 1.6 (95%CI 1.3–1.9; Table 3 (Tab. 3)). When compared to all BDs, DCWs showed higher positive prevalence rates in all age groups. However, when compared to the BDPcity subgroup, the prevalence rates in the different age groups were similar (Table 4 (Tab. 4)). Therefore, the subgroup comprising BDs with at least one pregnancy in their medical history and residing in the city of Hamburg had a prevalence rate similar to that of DCWs (53.9 vs 54.6%). The age-adjusted OR was 0.9 (95%CI 0.8–1.2) (no table).

## Discussion

We analyzed anti-CMV IgG seroprevalence datasets from pregnant DCWs and female BDs in the same region. The results for both groups were in the range of prevalence rates reported for European populations [9], [12]. As assumed, DCWs had a higher anti-CMV IgG prevalence than did the female BDs. BDs living in the metropolitan region of Hamburg had a higher CMV prevalence than BDs who lived in the wealthier suburbs of Hamburg (BDS). Our results therefore confirm the relevance of socioeconomic factors (SES) previously described by other authors [13].

However, when comparing DCWs with the best-matching subgroup of female BDs (BDPcity) with at least one pregnancy and residing in the city of Hamburg, no significant difference in prevalence was found, contradicting the hypothesis of an increasing risk during working life.

It is likely that anti-CMV IgG among pregnant DCWs is underestimated in our study because the specific IgG was only tested for DCWs with unknown or negative tests in their medical history. Furthermore, there was no information on the migration background (yes/no) of participants in either group. The dataset of DCWs working in Hamburg did not include information about the presence of children in the household or the postal code of current residence. Both datasets (DCWs and BDs) were convenience samples used for this secondary analysis; we therefore could not systematically examine risk factors such as private contact with young children, number of children in the household, or migrant status. 

Depending on the region and social background, the anti-CMV prevalence in adults ranges from 45 to 100% [16], [17], [18], [19]. Adler [20] observed a higher risk for DCWs who cared for children under two years of age compared to DCWs in charge of older children (SP 46 vs 35%; RR 1.29; 95%CI 1.05–1.57; p<0.02). DCWs had a significantly higher risk for seroconversion than did female hospital employees (RR 5.0; 95%CI 2.4–10.5; p<0.001). Moreover, most of the DNA patterns of isolates shed by children were identical to the patterns of the seroconverting DCWs. Ford-Jones et al. [21] confirmed a higher incidence among employees under the age of 30, working with infants, and changing diapers without using gloves. Infants shed viruses more often than toddlers (21% vs 8%, average 17%). Furthermore, Jones et al. [22] reported null CMV seroconversion but a higher seropositivity in DCWs in daycare centers for children with normal development than staff in centers for the developmentally delayed (60 vs 42%; p<0.05), depending on the SES of the children there and corresponding negative viral shedding rates (0 to 38%). Murph et al. [23] associated poor hygiene practices and new CMV shedding in children with a higher infection rate for DCWs (0 to 22% by 12 months; average 7.9%). Bale et al. [24] also described caring for children aged 1 to 2 years (p=0.02) as the strongest predictor of seropositivity. Summarizing the above-mentioned studies, Hyde et al. [19] reported a CMV seroconversion rate from 0 to 12.5% for North American DCWs until 1996 (summary annual infection rate = 8.5%; 95%CI 6.1–11.6%).

Pass et al. [25] were the first to observe a higher CMV risk for DCWs associated with employment or the demographic variable “contact with children younger than three years of age for at least 20 hours per week”. In Canada, Soto et al. [26] described a much higher seroconversion rate in a convenience sample of DCWs working with children younger than three years compared with other DCWs (50 vs 8%). Jackson et al. [27] found that only non-white ethnicities (OR 2.4; 95%CI 1.2–5.0; p=0.01), changing diapers three or more times per week (OR 1.8; 95%CI 1.1–2.8; p=0.02), and having a child living in the household (OR 1.8; 95%CI 1.1–2.9; p=0.01) had a significant impact. The evidence described above was summarized in five reviews [19], [28], [29], [30], [31] relating to nine US/Canadian papers [21], [20], [22], [23], [24], [25], [26], [27]. In addition, Joseph et al. [32] observed an elevated occupational risk at a child-to-educator ratio of more than six children of 18 to 35 months of age (OR 1.87; 95%CI 1.25–2.81). However, the occupational risk was lower than the personal risk of having two or more children of one’s own (OR 1.98; 95%CI 1.19–3.31). In summary, if the control group consisted of hospital workers who differ in several demographic features, or pregnant women, a comparison of rates would suggest an approximately five- to tenfold increase in the risk of CMV infection for DCWs in North America [25]. 

Current data revealed a decrease in CMV antibody prevalence [13], [14]. In 2002, a survey from Belgium examined the influence of hygiene measures for nursery school teachers in charge of children older than 2.5 years. The private risk (i.e., number of children at home; OR 2.25) was higher than the occupational risk (OR 1.54) [33]. In a French study, lifestyle factors were found to be as important as the occupational risk of a CMV infection [34]. However, in this study, no clear distinction could be made between those who were only caring for younger children and those DCWs in charge of older ones as well. A study from the Netherlands observed that the first two years of daycare employment posed a higher risk than later years (adjusted OR 3.80; p<0.001) [35]. Another study from the Netherlands showed an association between the country of birth and the prevalence of CMV IgG in DCWs (OR 1.7; 95%CI 1.3–2.3) [36]. 

Some evidence corroborates the assumption of a predominant risk factor “children under two or three years”. Children in DCCs were therefore examined. Twenty years ago, up to 50% of children attending DCCs shed CMV in Sao Paulo [11]. Recently, a French study found that children in DCCs were more likely to spread CMV than a control group comprising children in medical care (51.7 vs 21.7%) [37]. We did not find any data about CMV shedding rates in children in DCCs in Germany. 

Regarding evidence about the impact of personal hygiene, there is a broad consensus that direct contact with urine and saliva from young children must be avoided. Hand hygiene is crucial, as CMV is sensitive to soap and disinfectants [21], [23], [38], [33], [39], [40], [41]. As there is currently no vaccine available, hygiene interventions offer the best protection [17], [42]. 

## Conclusions

Our results show that half of DCWs – especially young women – are still at risk of a primary infection during pregnancy resulting in a risk of congenital CMV infection. Our data do not indicate an occupational risk of CMV infection among pregnant DCWs in Hamburg compared to female BDs. This observation is in line with results of recent studies. In addition, we are not aware of any well-designed studies examining the influence of appropriate hygiene in DCCs on CMV transmission. If it could be shown that hygiene measures effectively prevent CMV transmission to DCWs, it would be possible to relax job restrictions for pregnant DCWs.

## Notes

### Competing interests

The authors declare that they have no competing interests.

## Figures and Tables

**Table 1:**
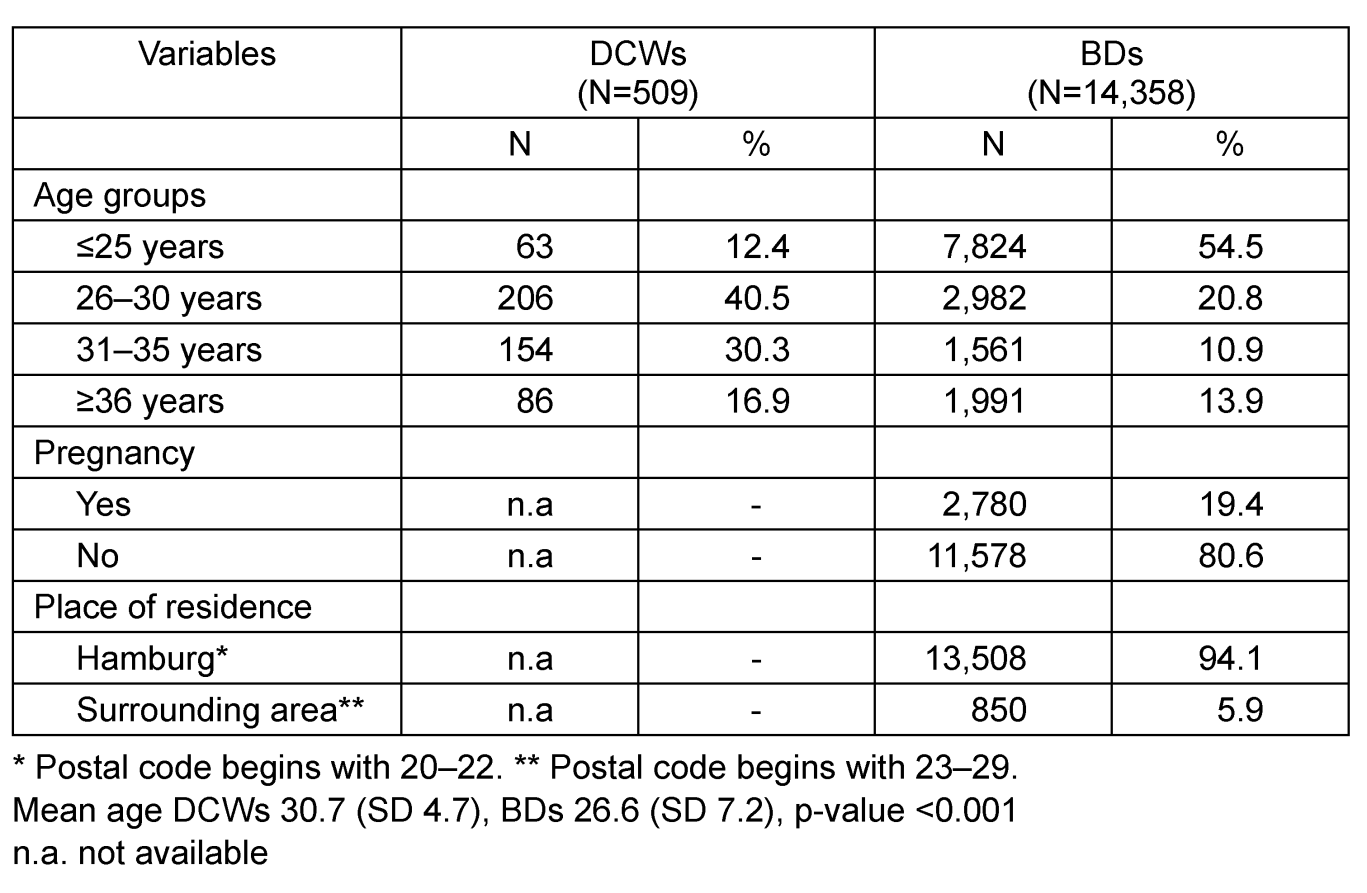
Characteristics of DCWs and BDs

**Table 2:**
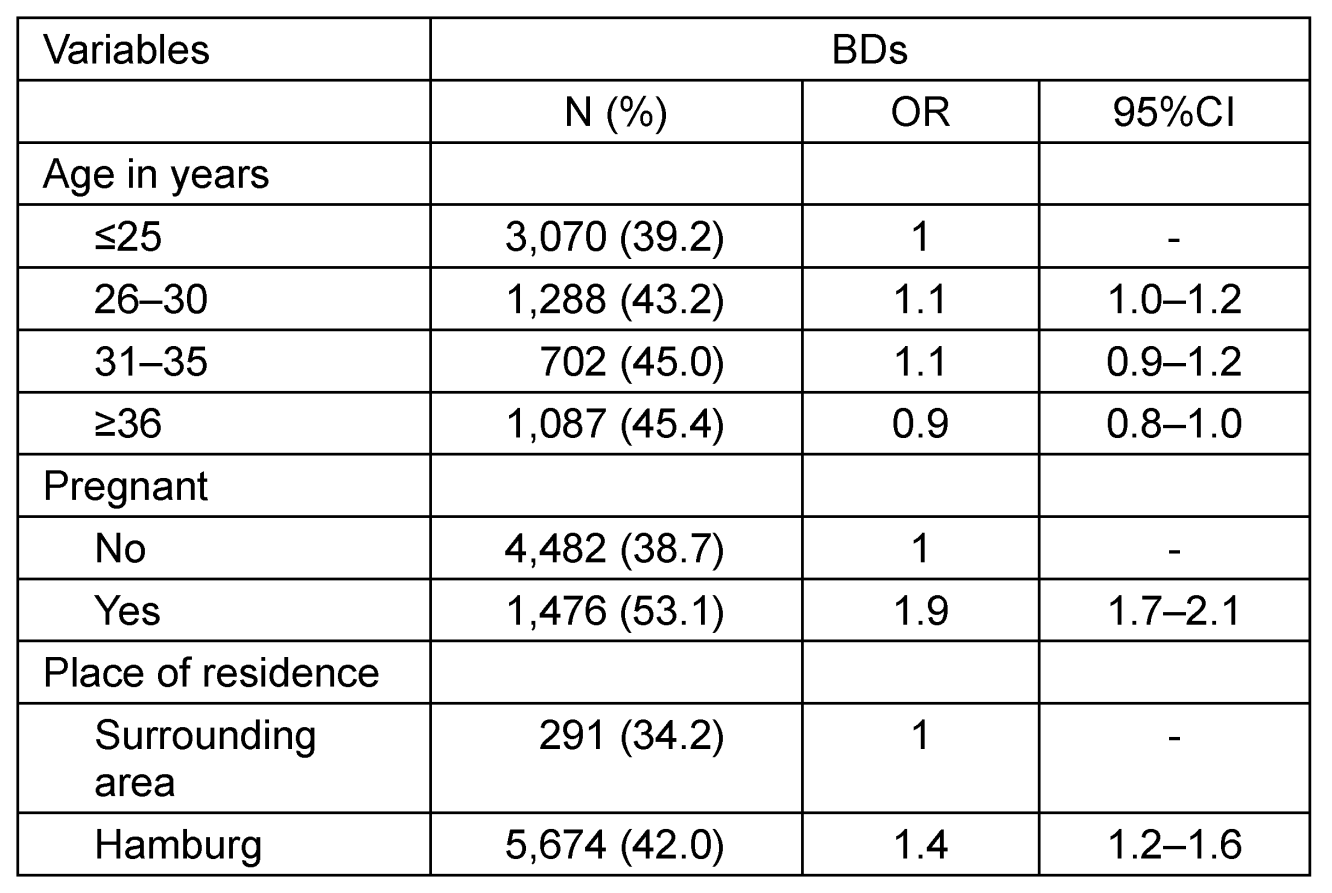
Adjusted odds ratios for anti-CMV IgG positivity by age, pregnancy, and place of residence among BDs

**Table 3:**
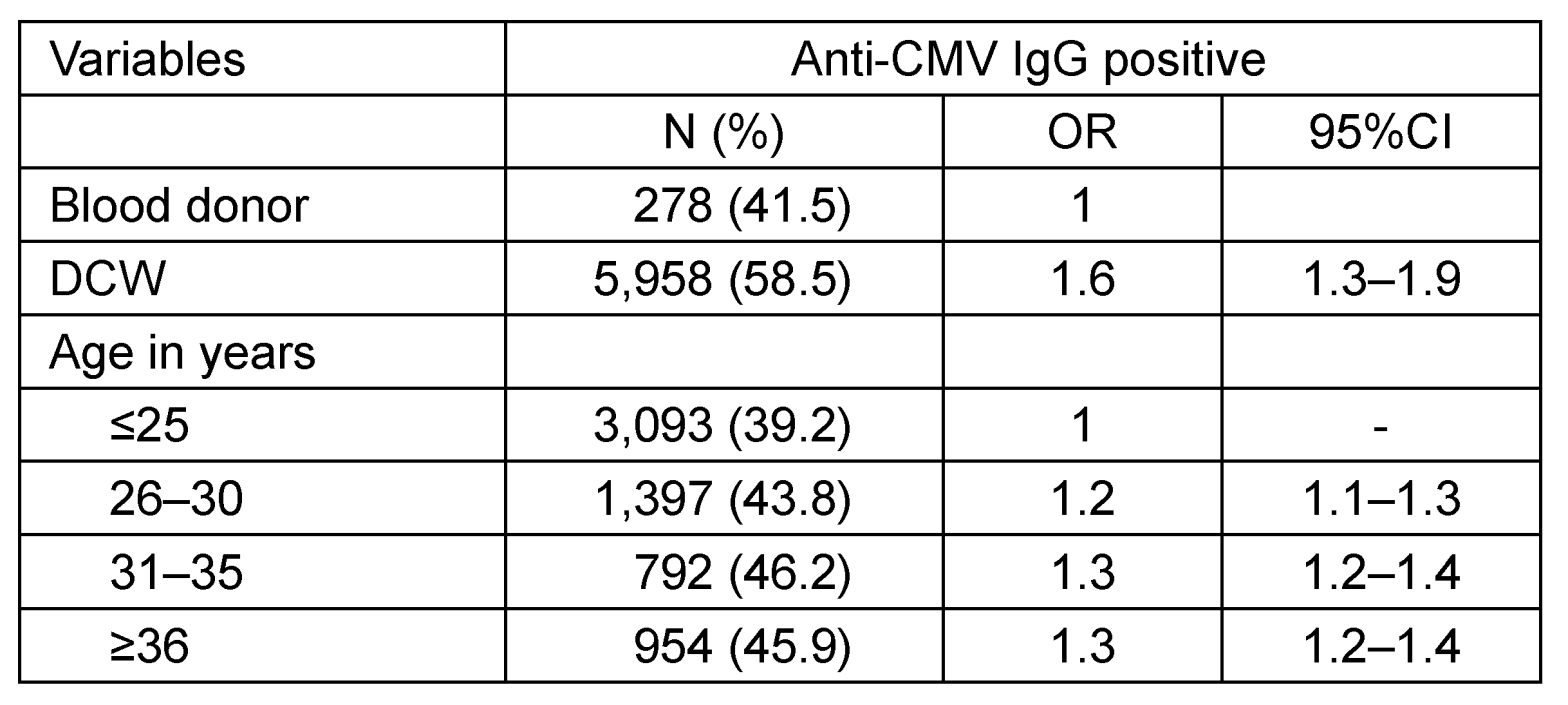
Adjusted odds ratios for anti-CMV IgG positivity depending on status as DCW and on age

**Table 4:**
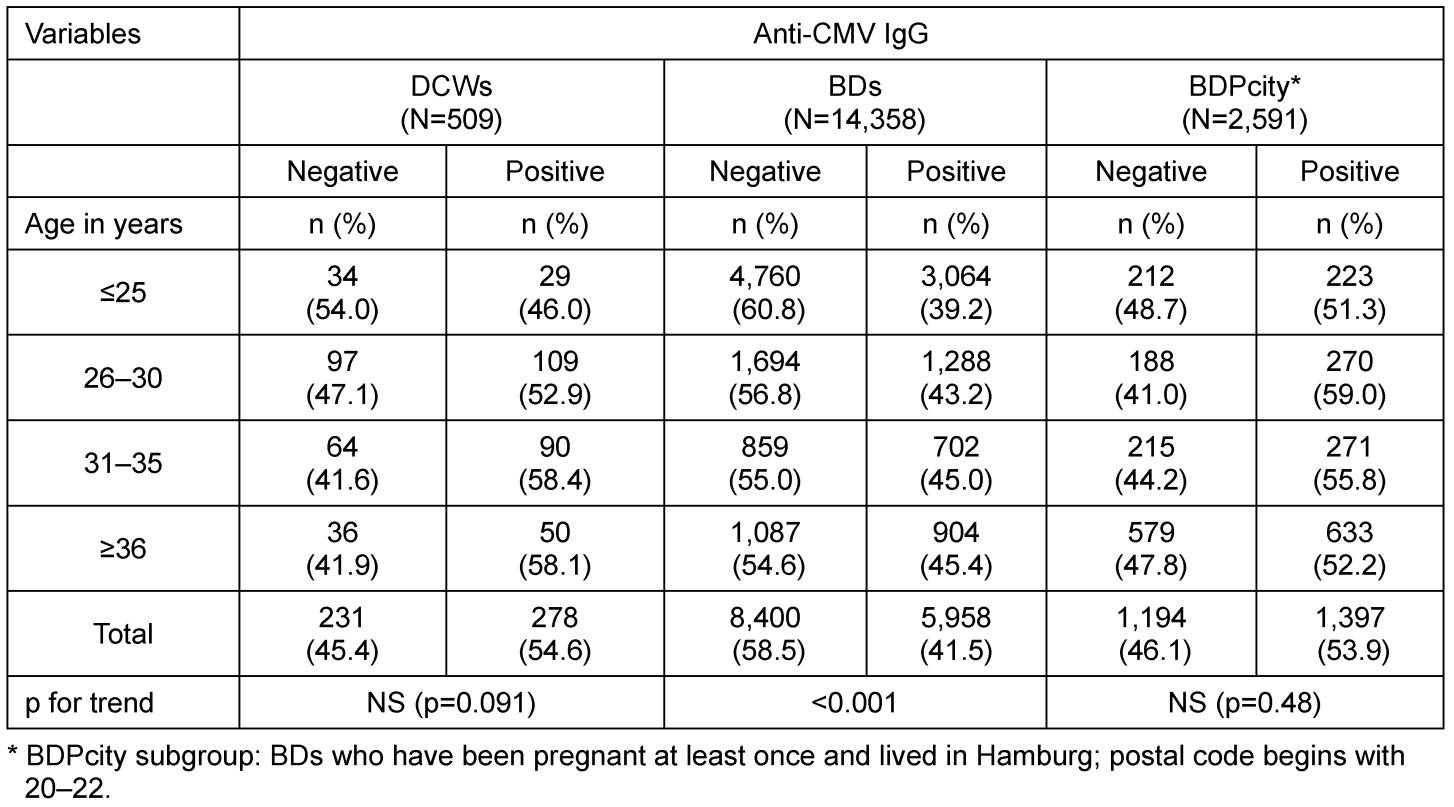
Anti-CMV IgG of DCWs, BDs, and subgroup (BDPcity) by age
